# The Predictive Value of *MAP2K1/2* Mutations on Efficiency of Immunotherapy in Melanoma

**DOI:** 10.3389/fimmu.2021.785526

**Published:** 2022-01-06

**Authors:** Ting Ye, Jie-Ying Zhang, Xin-Yi Liu, Yu-Han Zhou, Si-Yue Yuan, Meng-Mei Yang, Wen-Zhuan Xie, Chan Gao, Yao-Xu Chen, Meng-Li Huang, Cheng-Zhi Ye, Jing Chen

**Affiliations:** ^1^ Cancer Center, Union Hospital, Tongji Medical College, Huazhong University of Science and Technology, Wuhan, China; ^2^ The Medical Department, 3D Medicines Inc., Shanghai, China; ^3^ Department of Pediatrics, Renmin Hospital of Wuhan University, Wuhan, China

**Keywords:** melanoma, CTLA-4 blockade, PD-1 blockade, MAPK pathway, immunotherapy

## Abstract

**Background:**

*MAP2K1/2* genes are mutated in approximately 8% of melanoma patients; however, the impact of *MAP2K1/2* gene alterations on the efficiency of immunotherapy has not been clarified. This study focused on the correlation between *MAP2K1/2* gene mutations and the treatment response.

**Methods:**

Six metastatic melanoma clinical cohorts treated with immune checkpoint inhibitors [anti-cytotoxic T lymphocyte antigen-4 (CTLA-4) or anti-programmed cell death-1 (PD-1)] were recruited in this study. RNA expression profiling results from each of these six cohorts and the Cancer Genome Atlas (TCGA) melanoma cohort were analysed to explore the mechanism related to immune activation.

**Results:**

Compared to patients with wild-type *MAP2K1/2*, those with *MAP2K1/2* mutations in an independent anti-CTLA-4-treated cohort had higher objective response rates, longer progression-free survival, and longer overall survival (OS). These findings were further validated in a pooled anti-CTLA-4-treated cohort in terms of the OS. However, there was no correlation between *MAP2K1/2* mutations and OS in the anti-PD-1-treated cohort. Subgroup Cox regression analysis suggested that patients with *MAP2K1/2* mutations received fewer benefits from anti-PD-1 monotherapy than from anti-CTLA-4 treatment. Furthermore, transcriptome profiling analysis revealed that melanoma tumours with *MAP2K* mutation was enriched in CD8^+^ T cells, B cells, and neutrophil cells, also expressed high levels of CD33 and IL10, implying a potential mechanism underlying the benefit of melanoma patients with *MAP2K1/2* mutations from anti-CTLA-4 treatment.

**Conclusions:**

*MAP2K1/2* mutations were identified as an independent predictive factor for anti-CTLA-4 therapy in melanoma patients. Anti-CTLA-4 treatment might be more effective than anti-PD-1 therapy for patients with *MAP2K1/2-*mutated melanoma.

## Background

Treatment with immune checkpoint inhibitors (ICIs), including antibodies targeting cytotoxic T lymphocyte antigen-4 (CTLA-4) and programmed cell death-1/programmed cell death ligand 1 (PD-1/PD-L1), is becoming a novel therapeutic paradigm for melanomas (1). Ipilimumab, an anti-CTLA-4 monoclonal antibody, has been found to significantly improve overall survival (OS) and progression-free survival (PFS) and increase the long-term survival rate of patients with advanced melanoma (2, 3). Compared to ipilimumab, second-generation ICIs targeting PD-1, namely nivolumab and pembrolizumab, have also been reported to induce an increased response rate, OS, and PFS, with superior toxicity profiles (4, 5). Moreover, ICIs are the standard of care in the systemic treatment of metastatic or unresectable melanomas (6).

Although ICIs significantly increase the survival of melanoma patients, only a subset of patients can benefit from the therapy, and the related mechanisms are still not fully understood. Therefore, the identification of biomarkers to select patients who will be more responsive to ICIs is of utmost importance. Several genomic features, such as high mutational load, high neoantigen load, and tumour clonality have been found to be predictive of a favourable response to anti-CTLA-4 therapy in melanoma patients (7, 8). In addition, it has been reported that aberrations of individual genes, such as *SERPINB3/SERPINB4*, *NRAS*, and *TP53*, are associated with the response to anti-CTLA-4 therapy (9–11). Either high PD-L1 expression or high tumour mutational burden (TMB) has also been recognized as predictors of the effectiveness of anti-PD-1 blockade in melanomas and other solid tumours (12, 13).

The status of driver mutations may also influence the response to ICIs. For example, *NRAS*-mutated melanoma is a distinct subtype in approximately 5%–20% of patients with melanomas and appears to have a poor prognosis (12, 13). In a retrospective analysis, compared to melanoma patients with wild-type *NRAS*, those with *NRAS* mutations were found to have higher objective response rates and prolonged stable disease in response to ICIs (11). *NRAS* encodes N-Ras, which is a component of the Ras/Raf/MEK/ERK signalling cascade (14). This cascade, also known as the Ras/MAPK signalling cascade, plays an important role in the pathogenesis of melanoma (15). MEK is one of the kinases involved in the Ras/MAPK signalling cascade. Moreover, the encoding genes *MAP2K1* and *MAP2K2* (*MAP2K1/2*) are frequently mutated in melanoma, with a frequency of approximately 8% of cases (16, 17). The occurrence of *MAP2K1/2* mutations was identified as a mechanism related to BRAF inhibitor resistance in melanoma (18). To date, there are no reports on effective small molecule inhibitors targeting *MAP2K1/2* in melanoma. It is also unclear whether mutations in these genes influence the efficacy of immunotherapy.

Preclinical studies have revealed that treatment with MEK inhibitors might improve the sensitivity of immunotherapy in melanoma. MEK inhibition in a melanoma cell line was found to increase the antigen levels, which might potentiate anti-tumour T-cell immunity (19). Moreover, MEK inhibition may reduce the number of Bregs while sparing anti-tumour B-cell function, thereby enhancing anti-tumour immunity (20). In mouse models, MEK inhibitors were found to inhibit tumour growth *via* increasing the number of intertumoral effector-phenotype CD8^+^ T cells, and combination therapy with both MEK inhibitors and anti-PD-L1 agents exhibited a synergistic effect on antitumor growth (21).

In this study, through analysis of the sequencing and survival data for several public cohorts with metastatic melanoma, we investigated the association between *MAP2K1/2* mutations and the response to anti-CLTA-4 and anti-PD-1 immunotherapies. The influence of *MAP2K1/2* mutations on the expression of immunity-related genes was also evaluated by analysing the RNA expression profile data collected from these cohorts and from the Cancer Genome Atlas (TCGA) melanoma cohort. Our aim is to clarify the impact of *MAP2K1/2* gene alterations on the efficiency of immunotherapies and to provide guidance for treatment decision-making in *MAP2K1/2*-mutated melanomas.

## Methods

### Eligible Literature Search

We performed a systematic computerized search of the MEDLINE (PubMed) database and the Embases database up to November 1, 2020. The search terms were as follows: (Melanomas [MeSH] OR “metastatic melanomas” [Title/Abstract]) AND (“PD-1 blockade”[Title/Abstract] OR “PD-L1 blockade” [Title/Abstract] OR “CTLA‐4 blockade” [Title/Abstract] OR “immune checkpoint inhibitor” [Title/Abstract] OR “immune checkpoint inhibitors” [Title/Abstract] OR “ICI” [Title/Abstract] OR “ICIs” [Title/Abstract] OR “immune checkpoint blockade” [Title/Abstract] OR “immune checkpoint blockades” [Title/Abstract] OR “ICB” [Title/Abstract] OR “ICBs” [Title/Abstract]). Studies with eligible next-generation sequencing data were identified by hand and included if they met the criteria: (a) Clinical trials or study cohorts treated with ICIs; (b) Clinical outcomes of patients were available; (c) The number of evaluable patients was more than 30. We found six cohort studies of metastatic melanoma, specifically the Allen (8), Snyder (7), Hugo (22), and Liu (23) cohorts, and two metastatic pan-cancer cohorts comprising patients with melanoma, namely the Miao (24) and Samstein (25) cohorts.

### Study Design and Data Acquisition

In this study, missense, nonsense, and frame-shift mutations in both *MAP2K1* and *MAP2K2* genes were defined as *MAP2K1/2* mutations.

In total, data for 753 melanoma patients were included in our study. Notably, melanoma patients treated with sequential CTLA-4 and PD-1 blockades were excluded from this study. First, we determined the predictive value for CTLA-4 monotherapy of *MAP2K1/2* mutations in the Allen cohort and validated this predictive value in a CTLA-4-monotherapy-pooled cohort comprising 239 melanoma samples from the Snyder, Miao, and Samstein cohorts. Thereafter, we explored the impact of *MAP2K1/2* mutations on OS in a PD-1-monotherapy-pooled cohort consisting of 285 melanoma samples from the Hugo, Liu, Miao, and Samstein cohorts. The TCGA-skin cutaneous melanoma (SKCM) cohort without ICI treatment (n = 455) and with chemotherapy (n = 73) was analysed to assess the prognostic value of *MAP2K1/2* (**Figure S1**).

Data accessibility: data for whole-exome sequencing (WES), copy number alterations (CNAs), gene expression, and clinicopathologic information were collected from the Allen, Snyder, Hugo, and TCGA-SKCM cohorts. Data for targeted-sequencing and OS were obtained from the Samstein cohort using the cBioPortal database. Data for the Liu and Miao cohorts were taken from previous publications.

### Evaluation of Clinical Response

In evaluating the clinical response, responders were defined as melanoma patients with different degrees of clinical responses to immunotherapy, characterized as complete response (CR), partial response (PR), or stable disease (SD). Non-responders were defined as those patients with disease progression (PD) following immunotherapy.

### Transcriptome Data Normalization and Processing

The transcriptome data were interpreted as fragments per kilobase million mapped reads (FPKM). To compensate for RNA-seq counts within and between samples, the FPKM for every gene was transformed into transcripts per kilobase million (TPM) values by dividing by the sum of FPKM in each sample. The immune filtration cells scores were estimated with the TIMER2.0 website tool using TPM data.

### Statistical Analysis

In this study, all data analysis and graphic plotting were performed using the R software (v.3.6.3). Kaplan–Meier curves (log-rank test) of OS were plotted to compare survival outcomes between different subgroups. The proportion of death events of the patients in *MAP2K1/2*-mutated group and *MAP2K1/2*-wild-type groups was defined as risk ratio (RR). Pooled analysis was performed using the DerSimonian–Laird random-effects model to compare the RR with 95% of confidence intervals (CI) The degree of heterogeneity between cohorts was assessed *via* the *I* ^2^ index. No significant heterogeneity was defined as *I* ^2^ < 50% and P >0.1. Univariate and multivariate Cox proportional hazard regression analyses were performed to quantify the hazard ratio (HR) of various characteristics. Fisher’s exact tests were used to determine if there were non-random associations between categorical variables. The Wilcoxon–Mann–Whitney test was used to compare the differences in discrete ordinal data between two independent groups.

## Results

### *MAP2K1/2* Mutations as a Biomarker to Predict Favourable Response to Anti-CTLA-4 Therapy and Survival in Metastatic Melanoma

**Figure 1:**
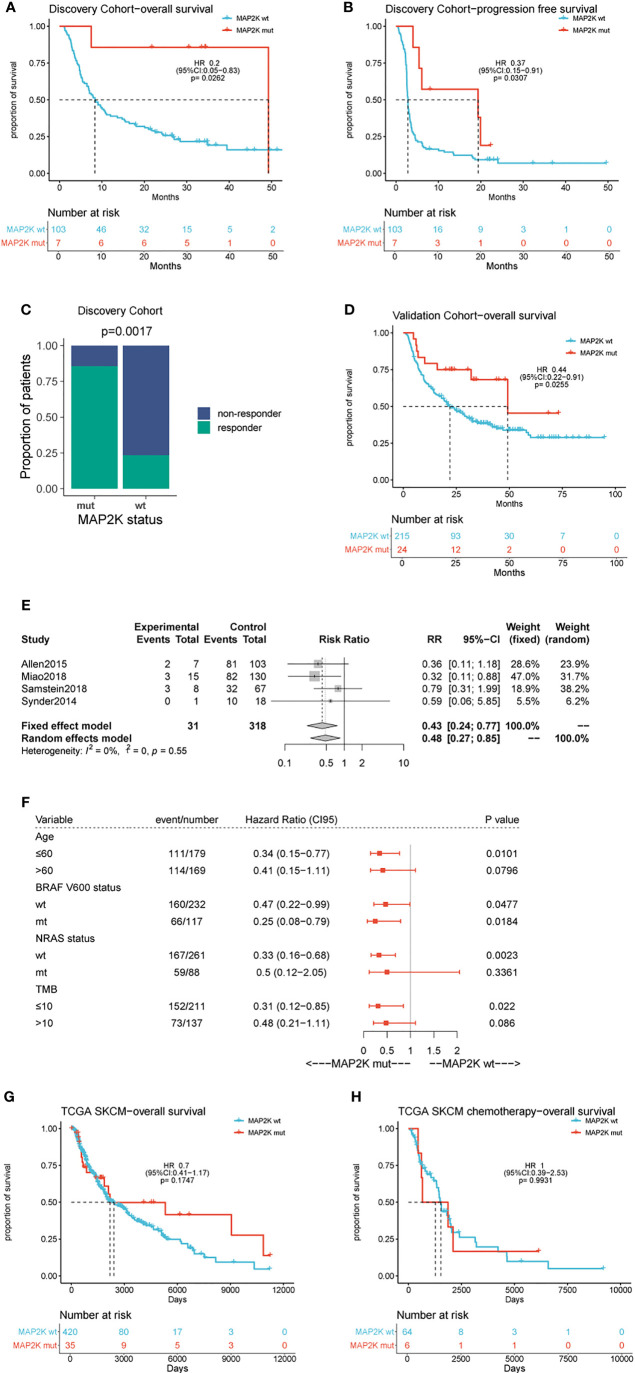
Effect of *MAP2K1/2* gene mutations on treatment response in anti-CTLA-4 and non-immunotherapy-treated melanoma. Kaplan-Meier analyses of overall survival (OS) **(A)**, progression-free survival (PFS) **(B)**, and disease-control rate **(C)** in the anti-CTLA-4-treated discovery cohort. Kaplan-Meier analyses of overall survival (OS) **(D)** in the anti-CTLA-4-treated validation cohort. Pooled estimates of OS in four anti-CTLA-4-treated cohorts **(E)**. Subgroup Cox analysis of OS in pooled anti-CTLA-4-treated cohorts among patients with and without MAP2K1/2 gene mutations **(F)**. Kaplan-Meier analyses of OS in TCGA melanoma cohort **(G)** and TCGA chemotherapy-treated cohort **(H)**.

Data analysis was performed using a cohort of 110 patients (Van Allen cohort) with metastatic melanoma treated with ipilimumab. The characteristics of the patients are summarized in **Table S1**. Seven patients (6.36%) in this cohort harboured *MAP2K1* or *MAP2K2* mutations and had longer OS (49.2 months vs 8.3 months; HR = 0.2; 95% confidence interval (CI), 0.05–0.83; p = 0.0262; **Figure 1A**) and PFS (19.4 months vs 2.8 months; HR = 0.37; 95% CI, 0.15–0.91; p = 0.0307; **Figure 1B**) than those with wild-type *MAP2K1/2*. Moreover, *MAP2K1/2* mutations were more frequent in responders (17.6% vs 1.3%; p = 0.0185; **Figure 1C**). In univariate analyses, three factors, namely *MAP2K1/2* mutations, tumour stage (stage 4 vs stage 3), and the presence of lactate dehydrogenase (LDH; 1 vs 0), were found to be associated with immunotherapeutic OS and PFS (**Table 1**). Furthermore, in the multivariable Cox proportional hazards regression model adjusted by tumour stage, LDH, and *MAP2K1/2* mutations, these three factors were still significantly associated with OS (HR = 0.24; 95% CI, 0.059–0.99; p = 0.048; **Table 1**) and PFS (HR = 0.39; 95% CI, 0.16–0.99; p = 0.048; **Table 1**). The results suggested that *MAP2K1/2* mutations might be an independent predictor for a favourable clinical response to anti-CTLA4 therapy in patients with melanoma.

**Table 1:** (a) Hazard ratio (HR) for OS *via* univariate and multivariate analyses in discovery cohort.

	Univariate		Multivariate	
Variable	HR (95% Cl)	P value (log-rank)	HR (95% Cl)	P value (log-rank)
Age (≤60 vs >60)	0.87 (0.57-1.35)	0.537		
Gender (male vs female)	0.78 (0.49-1.24)	0.301		
Stage (Stage 4 vs Stage 3)	4.58 (1.44-14.56)	0.001	3.76 (1.18-12)	0.025
LDH (1 vs 0)	2.07 (1.33-3.22)	0.001	2.04 (1.31-3.18)	0.002
BRAF V600 status (mut vs wt)	0.7 (0.43-1.16)	0.153		
NRAS status (mut vs wt)	1.14 (0.69-1.9)	0.610		
TMB (>median vs ≤median)	0.72 (0.47-1.11)	0.143		
TMB (top 20% vs bottom 80%)	0.73 (0.42-1.27)	0.252		
MAP2K status (mut vs wt)	0.2 (0.05-0.83)	0.004	0.24 (0.059-0.99)	0.048

**Table 1:** (b) Hazard ratio (HR) for PFS *via* univariate and multivariate analyses in discovery cohort.

	Univariate		Multivariate	
Variable	HR (95% Cl)	P value (log-rank)	HR (95% Cl)	P value (log-rank)
Age (≤60 vs >60)	0.97 (0.65-1.44)	0.889		
Gender (male vs female)	0.93 (0.6-1.43)	0.735		
Stage (Stage 4 vs Stage 3)	2.41 (1.11-5.21)	0.012	1.83 (0.83-4.01)	0.025
LDH (1 vs 0)	2.04 (1.36-3.07)	0.001	2.05 (1.36-3.09)	<0.001
BRAF V600 status (mut vs wt)	0.87 (0.56-1.35)	0.53		
NRAS status (mut vs wt)	0.97 (0.61-1.55)	0.9		
TMB (>median vs ≤median)	0.86 (0.58-1.27)	0.442		
TMB (top 20% vs bottom 80%)	0.98 (0.6-1.59)	0.935		
MAP2K status (mut vs wt)	0.37 (0.15-0.91)	0.012	0.39 (0.16-0.99)	0.048

To validate the predictive value of *MAP2K1/2* mutations for the efficacy of anti-CTLA4 therapy in melanoma, data analysis was also performed using a pooled cohort, which was a combination of three metastatic melanoma cohorts treated with ipilimumab (Miao, Samstein, and Snyder) (**Table S1**). This pooled cohort contained 239 patients, among which 24 patients (10%) harboured *MAP2K1* or *MAP2K2* mutations. According to the data analysis results, *MAP2K1/2* mutations were significantly correlated with longer OS in this pooled cohort (49.3 months vs 22.0 months; HR = 0.44; 95% CI, 0.22–0.91; p = 0.0255; **Figure 1D**) compared to the group with wild-type *MAP2K1/2*. The meta-analysis also demonstrated that the group with *MAP2K1/2* mutations exhibited a significantly reduced risk of death, as compared to the group with wild-type *MAP2K1/2* (fixed effects model; relative risk (RR) = 0.43; 95% CI, 0.24–0.77; **Figure 1E**). No substantial heterogeneity was observed across studies (p = 0.55, **Figure 1E**), indicating the conclusion was consistent, from data analysis between different cohorts to the association between *MAP2K1/2* mutations and the favourable clinical response to anti-CTLA-4 therapy. Further data analysis using a pooled cohort consisting of four cohorts also confirmed that *MAP2K1/2* mutations are a predictive factor for superior OS in most subgroups with diverse clinical and molecular characteristics (**Figure 1F**).

To clarify whether *MAP2K1/2* mutations are a predictive or prognostic biomarker, analysis of the data obtained from the TCGA database was performed, based on the *MAP2K1/2* mutational status of melanoma patients. No significant difference in OS was observed between the groups with mutated and wild-type *MAP2K1/2* in the total population (HR = 0.7; 95% CI, 0.41–1.17; p = 0.1747; **Figure 1G**) as well as in the chemotherapy population (HR = 1, 95% CI, 0.39–2.53; p = 0.99; **Figure 1H**). Taken together, these results suggested that *MAP2K1/2* gene mutations are a predictor for a favourable clinical response to anti-CTLA-4 therapy in metastatic melanoma rather than a prognostic factor for melanoma.

### *MAP2K1/2* Mutations Were Not Associated With the Clinical Benefits of Anti-PD-1/L1 Therapy for Metastatic Melanoma

**Figure 2:**
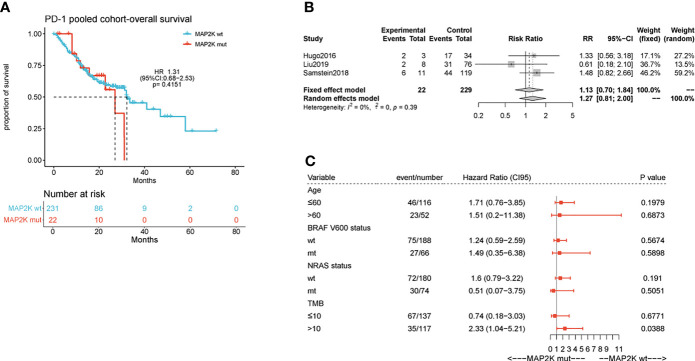
Effect of *MAP2K1/2* gene mutations on treatment response in anti-PD-1-treated melanoma. Kaplan-Meier analyses of overall survival (OS) in the anti-PD-1-treated cohort **(A)**. Pooled estimates of OS in three anti-PD-1-treated cohorts **(B)**. Subgroup Cox analysis of OS in pooled anti-PD-1 treated cohorts among patients with and without *MAP2K1/2* gene mutations **(C)**.

Anti-PD-1 therapy with second-generation ICIs can prolong both PFS and OS in metastatic melanoma patients, with less high-grade toxicity than ipilimumab. To investigate whether *MAP2K1/2* mutations are also associated with a favourable clinical response to anti-PD-1/L1 therapy, we performed data analysis using a pooled cohort consisting of three public cohorts of 253 metastatic melanoma patients treated with anti-PD-1 agents (**Table S1**). Overall, 22 patients (8.7%) harboured *MAP2K1/2* mutations in this pooled cohort. The results showed that there was no significant difference in OS between mutated and wild-type *MAP2K1/2* groups (27.0 months vs 32.0 months; HR = 1.31; 95% CI, 0.68–2.53; p = 0.4151; **Figure 2A**). The meta-analysis showed that the risk of death was not reduced in the *MAP2K1/2*-mutated group as compared to the wild-type *MAP2K1/2* group (fixed effects model; RR = 1.13; 95% CI, 0.7–1.84; **Figure 2B**). Moreover, subgroup analysis indicated that *MAP2K1/2* mutations were not a predictor of OS in any subgroup (**Figure 2C**). These results revealed that *MAP2K1/2* gene mutations are not associated with the clinical benefits of anti-PD-1/L1 therapy for metastatic melanoma. Therefore, the predictive value of *MAP2K1/2* mutation in metastatic melanoma might be specific to anti-CTLA-4 therapy, rather than anti-PD-1/L1 therapy.

### Mutation Status of *MAP2K1/2* Gene May Influence Systemic Treatment Options in Metastatic Melanoma

**Figure 3:**
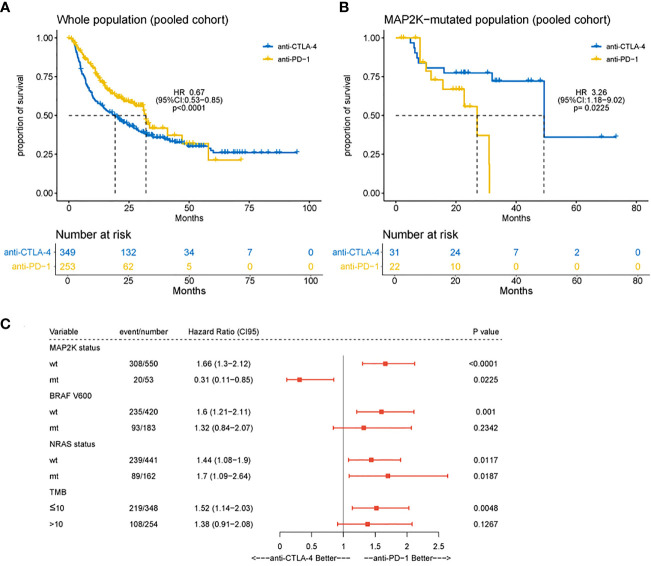
Difference in overall survival between melanoma patients receiving anti-CTLA-4 or anti-PD-1 monotherapy. Kaplan-Meier analyses of overall survival (OS) in overall population **(A)** and *MAP2K1/2*-mutated subgroup **(B)** in the combined cohort of anti-CTLA-4 and anti-PD-1-treated patients. Subgroup Cox analysis of OS among patients receiving anti-CTLA-4 or anti-PD-1 monotherapy **(C)**.

In this study, the clinical benefits (OS) of anti-CTLA-4 and anti-PD-1 therapies in patients with *MAP2K1/2*-mutated melanoma were compared in a pooled cohort comprising a combination of six cohorts. Analysis results showed that in the total population, the association of OS with anti-PD-1 therapy was superior to the association with anti-CLTA-4 therapy (median OS, 32.0 months vs 19.2 months; HR = 0.67; 95% CI, 0.53–0.85; p = 0.0009; **Figure 3A**), consistent with the results of a previous study (5). However, patients harbouring *MAP2K1/2* mutations treated with anti-PD-1 monotherapy had significantly poorer OS than those treated with anti-CTLA-4 monotherapy (median OS, 27.0 months vs 49.3 months; HR = 3.26; 95% CI, 1.18–9.02; p = 0.0225; **Figure 3B**). The difference in OS between the two immunotherapies could be attributed to the remarkably improved survival rate in the *MAP2K1/2*-mutated group treated with anti-CTLA-4 therapy. Subgroup analysis also revealed that the *MAP2K1/2*-mutated group was more likely to obtain clinical benefits from anti-CTLA-4 monotherapy, as compared to anti-PD-1 monotherapy (HR = 0.31; 95% CI, 0.11−0.85; **Figure 3C**), suggesting that for *MAP2K1/2*-mutated melanomas, the efficacy of anti-CTLA-4 therapy might be superior to that of anti-PD-1 therapy.

### Immunological Microenvironment of *MAP2K1/2*-Mutated Melanoma

**Figure 4:**
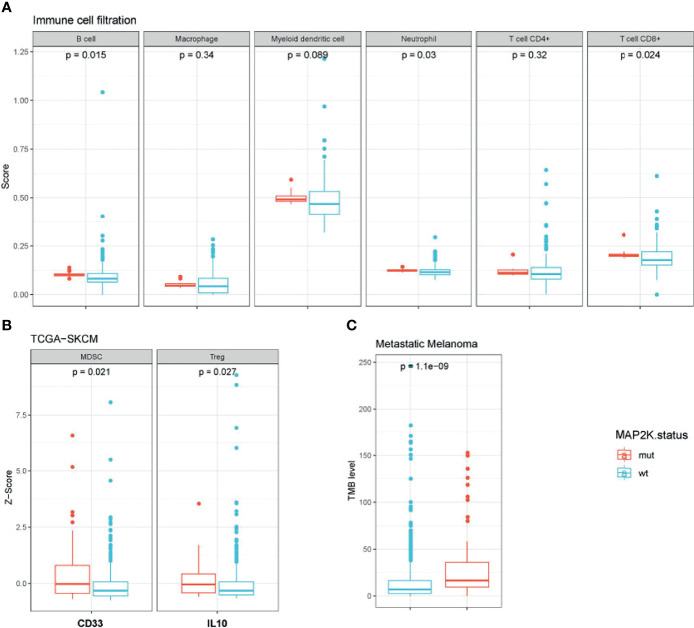
Immunological microenvironment of *MAP2K1/2*-mutated melanoma. Boxplot comparing immune cell filtration between mutated and wild-type *MAP2K1/2* subgroup in metastatic melanoma cohort **(A)**, the expression of immune-related genes between the mutated and wild-type *MAP2K1/2* subgroups in TCGA-SKCM cohort **(B)**, and tumour mutational level between mutated and wild-type *MAP2K1/2* subgroup in metastatic melanoma cohort **(C)**.

To evaluate the impact of *MAP2K1/2* mutations on the transcription of immunity-related genes in melanoma, we integrated and analysed the gene expression data for patients from four clinical cohorts and the TCGA-SKCM cohort. To investigate the status of immune cell infiltration in melanoma patients treated with immunotherapies, TIMER2.0, a comprehensive resource for systematic analysis of immune infiltrates across diverse cancer types, was used to analyse the gene expression data of *MAP2K1/2*-mutated melanoma. Analysis results revealed that *MAP2K1/2*-mutated melanoma exhibited significantly increased densities of B cells (p = 0.015), CD8^+^ T cells (p = 0.024), and neutrophils (p = 0.03) and a numerically higher level of myeloid dendritic cells (p = 0.089) compared to those in their wild-type counterparts, implying that melanoma patients with *MAP2K1/2* mutations have a favourable microenvironment for tumoral development (**Figure 4A**). However, there is no difference in macrophages or CD4^+^ T cells between *MAP2K1/2*-mutated and wild-type melanomas (**Figure 4A**). Interestingly, the gene expression levels of CD33, a marker of myeloid-derived suppressor cells (MDSCs), and IL-10, which is mainly secreted by regulatory T cells (Tregs), were higher in *MAP2K1/2*-mutated melanoma, compared to those in wild-type melanoma (**Figure 4B**). Moreover, MDSCs and Tregs were reported to be associated with resistance to the PD-1 blockade (26, 27), consistent with the poor prognosis of patients with *MAP2K1/2*-mutated melanoma who received anti-PD-1 therapy. TMB analysis also revealed that melanoma patients harbouring *MAP2K1/2* mutations have a higher level of TMB (**Figure 4C**). Moreover, except for immunity-related genes, a total of 55 significantly differentially expressed genes were found between *MAP2K1/2*-mutated and wild-type melanoma in clinical cohort (**Table S2**), which might be associated with reshape of immunological microenvironment caused by MAPK mutation.

## Discussion

In recent years, immunotherapy has greatly improved the survival and quality of life for patients with melanoma, becoming one of the standard treatment regimens for metastatic or advanced melanoma. Although various molecules/antigens have been proposed as possible immunotherapy targets, only anti-CTLA4 antibody and anti-PD-1/L1 antibody are available for immunotherapy against melanoma in clinical practice. A large-scale phase III clinical trial (CheckMate 066) confirmed the efficacy of the anti-PD-1 antibody nivolumab in melanoma treatment. The results showed that nivolumab treatment was superior to standard chemotherapy as the first-line treatment with respect to the OS, PFS, and overall response rate. Later, another trial (Keynote-006) reported the superiority of pembrolizumab to ipilimumab. These studies laid the foundation for the approval of immunotherapy as the first-line treatment for melanoma by the Food and Drug Administration (FDA). Nevertheless, immunotherapy for melanoma treatment is still limited by the fact that only a portion of patients receive the clinical benefits of immunotherapy. For patients who do not respond to immunotherapy, this issue may lead to unnecessary costs (a heavy financial burden for the family) and loss of a valuable period for tumour treatment. Therefore, the identification of biomarkers for predicting the efficacy of immunotherapy would be of great importance.

Among potential biomarkers, CTLA-4 and PD-1 are the leading targets of ICIs in cancer immunotherapy. Previous studies have shown that CTLA-4 and PD-1 have distinct signalling pathways related to the different action mechanisms of immunotherapy (28). Anti-CTLA-4 therapy primarily interferes with the feedback mechanism to improve the proliferation and activation of more T cells, while anti-PD-1 treatment is assumed to attenuate the tumour-induced immunosuppression (29). Retrospective studies have identified different biomarkers that can predict the efficacy of anti-CTLA-4 or anti-PD-1 therapy. For example, loss of major histocompatibility complex (MHC) class I expression is a predictor of resistance to anti-CTLA-4 but not to anti-PD-1 therapy. In contrast, the expression of MHC class II, which is associated with interferon-γ-related signatures, can predict the treatment response to anti-PD-1 therapy, rather than anti-CTLA-4 therapy (23, 28, 30). To further identify more novel predictive biomarkers, we performed data analysis using several public cohorts and found that *MAP2K1/2* mutations might be a potential predictor for the clinical benefits of anti-CTLA-4 therapy in advanced melanomas. Our study revealed that patients with *MAP2K1/2* mutations had longer PFS and OS than their counterparts without mutations when both groups of patients received ipilimumab treatment. However, the predictive value of *MAP2K1/2* mutations is specific to anti-CTLA-4 therapy, rather than anti-PD-1 treatment, because no difference in the survival rate was observed between patients with *MAP2K1/2* mutations and their wild-type counterparts after anti-PD-1 treatment. Based on these observations, we investigated the possibility that the difference in clinical benefits between anti-PD-1/L1 and anti-CTLA-4 therapies in patients with *MAP2K1/2*-mutated melanoma can be used as a biomarker for the selection of the appropriate immunotherapy drug. According to this proposed hypothesis, further data analysis was conducted to compare the efficacy of anti-PD-1/L1 and anti-CTLA-4 therapies in patients with *MAP2K1/2*-mutated melanoma. Our results indicated that patients with *MAP2K1/2*-mutated melanoma who received anti-CTLA-4 therapy had better OS. In other words, anti-CTLA-4 therapy was superior to anti-PD-1/L1 therapy for patients with *MAP2K1/2*-mutated melanoma.

Compared with other proposed efficacy indicators of immunotherapy, such as TMB, microsatellite instability (MSI), and PD-L1 expression, the use of *MAP2K1/2* gene mutations as a qualitative biomarker could avoid the dilemma of setting cut-off values. In addition, *MAP2K1/2* mutations can be detected in peripheral blood ctDNA, providing a non-invasive approach to identify patients who may receive the benefits of ICI treatment.

In addition, we explored the mechanism underlying the predictive role of *MAP2K1/2* in the clinical response to ICIs. TMB, defined as the number of somatic mutations per megabase of interrogated genomic sequences, is considered to be related to the outcome of ICI treatment across multiple tumour types (31–33), though the exact mechanism remaining controversial (34). We found that *MAP2K1/2*-mutated melanomas exhibited higher TMB levels than *MAP2K1/2*-wild-type melanomas. This phenomenon could be associated with the predictive effect of TMB on anti-CTLA-4 monotherapy, which was previously reported (7). In addition, we observed an increase in B cells, CD8^+^ T cells, and neutrophils in *MAP2K1/2*-mutated melanomas, as compared to *MAP2K1/2*-wild-type melanomas. Like dendritic cells, B cells can internalize antigens and deliver antigenic peptides to T-cell receptors (35). A previous study showed that high expression of immune cell-derived gene expression signatures in B cells is associated with better response to the anti-CTLA-4 antibody (36). Additionally, the number of MDSCs and Tregs is increased in *MAP2K1/2*-mutated melanomas. MDSCs are involved in immunosuppression *via* suppressing the functions of T-cells and natural killer-cells (27). It has been reported that MDSCs can induce the expansion of Tregs and reduce the anti-tumour activity of effector T cells (37), while Tregs can regulate immunosuppression by secreting cytokines, such as IL10, IL35, and TGF-β, thereby suppressing the effector T-cell response. These processes might account for the superiority of anti-CTLA-4 therapy over anti-PD-1 therapy in *MAP2K1/2*-mutated melanomas. Contrary to our expectations, there was no difference in the expression of MHC class I and II molecules between the *MAP2K1/2*-mutated and *MAP2K1/2*-wild-type groups (**Figures S2, S3**). For 55 significantly differentially expressed genes between MAP2K1/2-mutated and wild-type melanoma in clinical cohort, 14 genes (SMAD9, LRP6, PCDH18, TP53BP2, KDM1A, PKLR, GALNT5, RASGRF2, CTSK, ZNF845, ZNF384, TEK, MTHFD1, TAX1BP1) were associated with the immunological microenvironment as previous reports. Interestingly, TP53BP2, one of the 14 genes, has been proved to activate CD4+ and CD8+ immune and negatively regulate the MAPK signaling pathway in triple-negative breast cancers (38). The impact of *MAP2K1/2* gene mutations on the immunological microenvironment should be further assessed through *in vitro* or *in vivo* models. *MAP2K1/2*-mutated cases constitute approximately 8% of all melanoma patients, and the clinical studies of this population has been rarely reported. In fact, the inhibitory drugs of *MAP2K1/2*, more commonly called MEK inhibitors were identified to be effective in the treatment of melanomas. The combination of MEK inhibitor with BRAF inhibitor has become the standard of care for patients with *BRAF*-mutated melanoma (39–41). Moreover, in *NRAS*-mutated melanoma patients, binimetinib has shown its treatment efficacy and represents a treatment option after failure of immunotherapy (42). It has been reported that previous treatment with BRAFi with or without MEKi result in shorter survival in *BRAF-*mutated melanoma patients when treated with anti-PD-1 antibody (43). Nevertheless, the influence of previous targeted therapies on the therapeutic effect of immunotherapy in *MAP2K1/2*-mutated melanoma is unknown. Since no effective targeted therapeutic drugs have been reported against *MAP2K1/2*-mutated melanomas, ICIs are still considered as the preferred systemic treatment for these patients. To the best of our knowledge, the present manuscript is the first report on the investigation of the association between MAP2K1/2 mutations and the clinical response to ICIs. This study can also provide a guideline for making treatment decisions for patients with *MAP2K1/2*-mutated melanoma. With an innovation-based view, we suggest that for patients with *MAP2K1/2*-mutated melanoma, anti-CTLA-4 therapy might be more effective than anti-PD-1 monotherapy. Due to the lack of available data, we could not compare the differences in the efficacy of anti-CTLA-4 monotherapy and combination therapy involving both anti-CTLA-4 anti-PD-1 in patients with *MAP2K1/2*-mutated melanoma in this study. Because of the possibility that combination therapy may increase the incidence of grade 3–4 immune-related adverse events, it would be especially meaningful for patients with poor physical conditions if a comparison study is performed for benefit-risk assessment in ICIs to determine the optimal benefit/risk potential for patients.

There are still some limitations in this study. Because the ICI-treated cohorts included in this study came from several research centres, analysis of the data from the pooled-cohort might introduce biases due to differences in the ICI regimen, dose usage, and treatment cycle between institutions. Additionally, there was no specific limitation on the number of previous therapies used in the observed cohorts, which may cause heterogeneity in the survival time and might account for the OS benefit in the whole population not being as obvious as that reported in the phase III clinical trials, although the trend was consistent (44, 45). Because of the limited sample size and lack of molecular information, we could not match the baseline characteristics such as PD-L1 expression and microsatellite stability. Therefore, prospective studies are additionally required to confirm the findings of this study. Further studies are also needed to elucidate the mechanisms underlying the clinical benefits of anti-CTLA-4 therapy in *MAP2K1/2*-mutated melanoma.

## Data Availability Statement

The data of the Allen cohort, Snyder cohort, Hugo cohort, Liu cohort, Miao cohort and Samstein cohort included in this analysis were provided online. The specific accession numbers and repository names are included in **Supplementary Table 1**.

## Author Contributions

Conceptualization: C-ZY and JC. Methodology: M-LH. Validation: X-YL, W-ZX, and Y-XC. Investigation: M-MY. Data curation: Y-HZ and S-YY. Writing—original draft preparation: TY and J-YZ. Writing—review and editing: CG. All authors have read and agreed to the published version of the manuscript.

## Funding

This work was supported by the National Natural Science Foundation of China (81773285 to JC), the Scientific Research Project of Hubei Provincial Health and Family Planning Commission, China (WJ2015MB017 to JC), and the Health Commission of Hubei Province scientific research project, China (WJ2021Z004 to JC).

## Conflict of Interest

X-YL, M-MY, W-ZX, CG, Y-XC, and M-LH were employed by 3D Medicines Inc.

The remaining authors declare that the research was conducted in the absence of any commercial or financial relationships that could be construed as a potential conflict of interest.

## Publisher’s Note

All claims expressed in this article are solely those of the authors and do not necessarily represent those of their affiliated organizations, or those of the publisher, the editors and the reviewers. Any product that may be evaluated in this article, or claim that may be made by its manufacturer, is not guaranteed or endorsed by the publisher.

## Abbreviations

MAP2K, mitogen-activated protein kinase kinase; MAPK, mitogen-activated protein kinase; CTLA-4, cytotoxic T lymphocyte antigen-4; PD-1/PD-L1, programmed cell death-1/ligand-1; ICB, immune checkpoint blockade; ICIs, immune checkpoint inhibitors; CNAs, copy number alterations; WES, whole-exome sequencing; TMB, tumour mutational burden; MSI, microsatellite instability; SKCM, skin cutaneous melanoma; TCGA, The Cancer Genome Atlas; MSKCC, memorial Sloan Kettering cancer center; OS, overall survival; PFS, progression-free survival; CR, complete response; PR, partial response; SD, stable disease; PD, progressive disease.
