# Supplementary figures and images for: The Predictive Value of MAP2K1/2 Mutations on Efficiency of Immunotherapy in Melanoma
Source: Front Immunol. 2022 Jan 6;12:785526. doi: 10.3389/fimmu.2021.785526 (PMC8770828; doi:10.3389/fimmu.2021.785526)

**Figure S1**

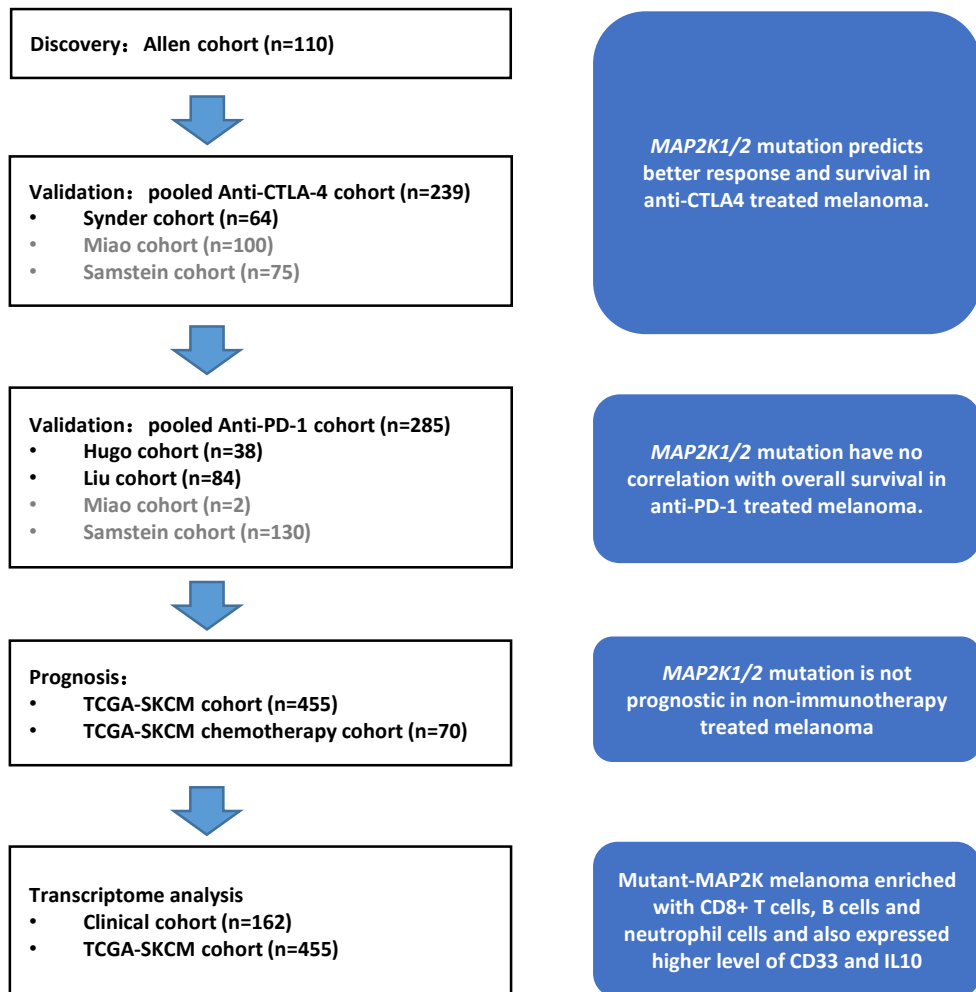

Supplement: Supplementary file 1 [file DataSheet_1.pdf]

Figure S2

**A**

metastatic melanoma

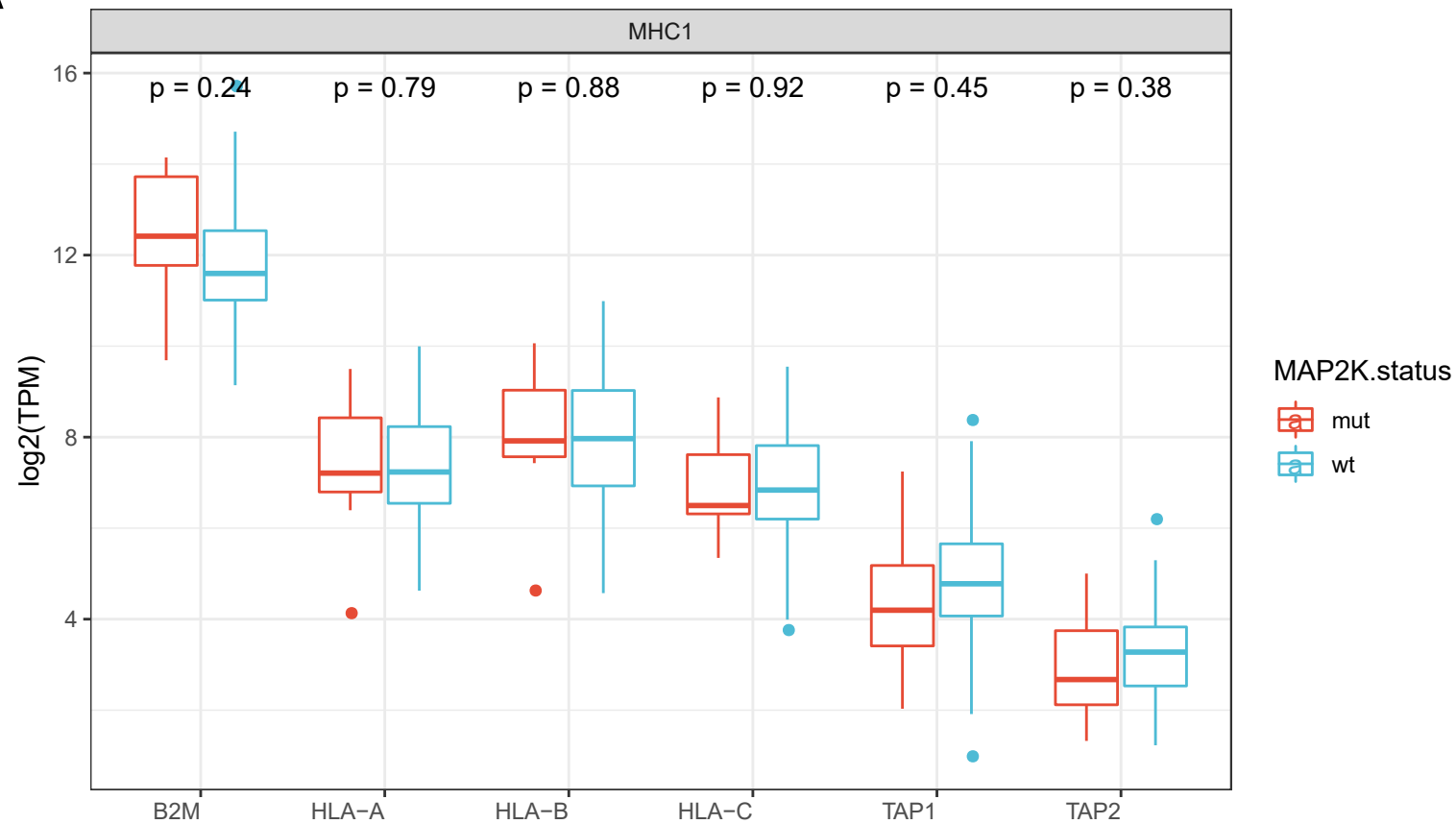

**B**

metastatic melanoma

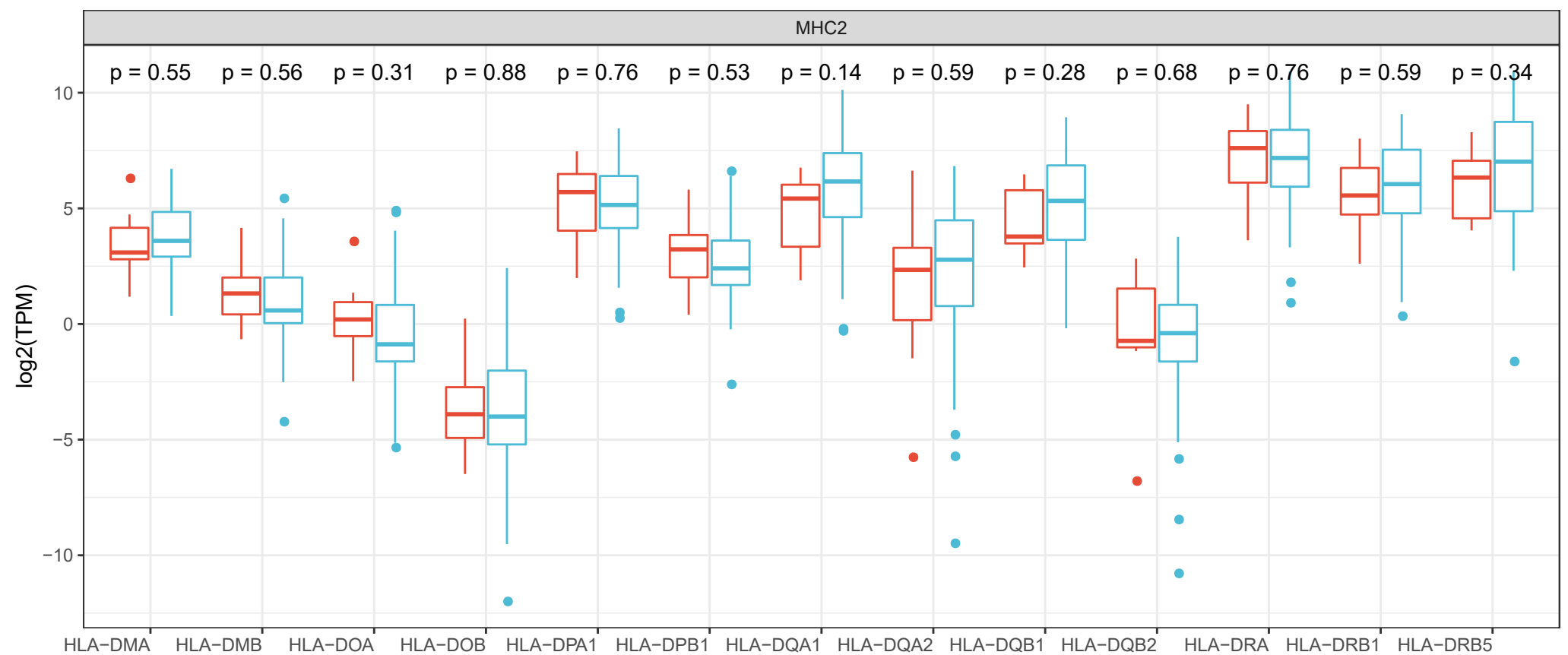

Supplement: Supplementary file 2 [file DataSheet_2.pdf]

Figure S3

**A**

TCGA-SKCM

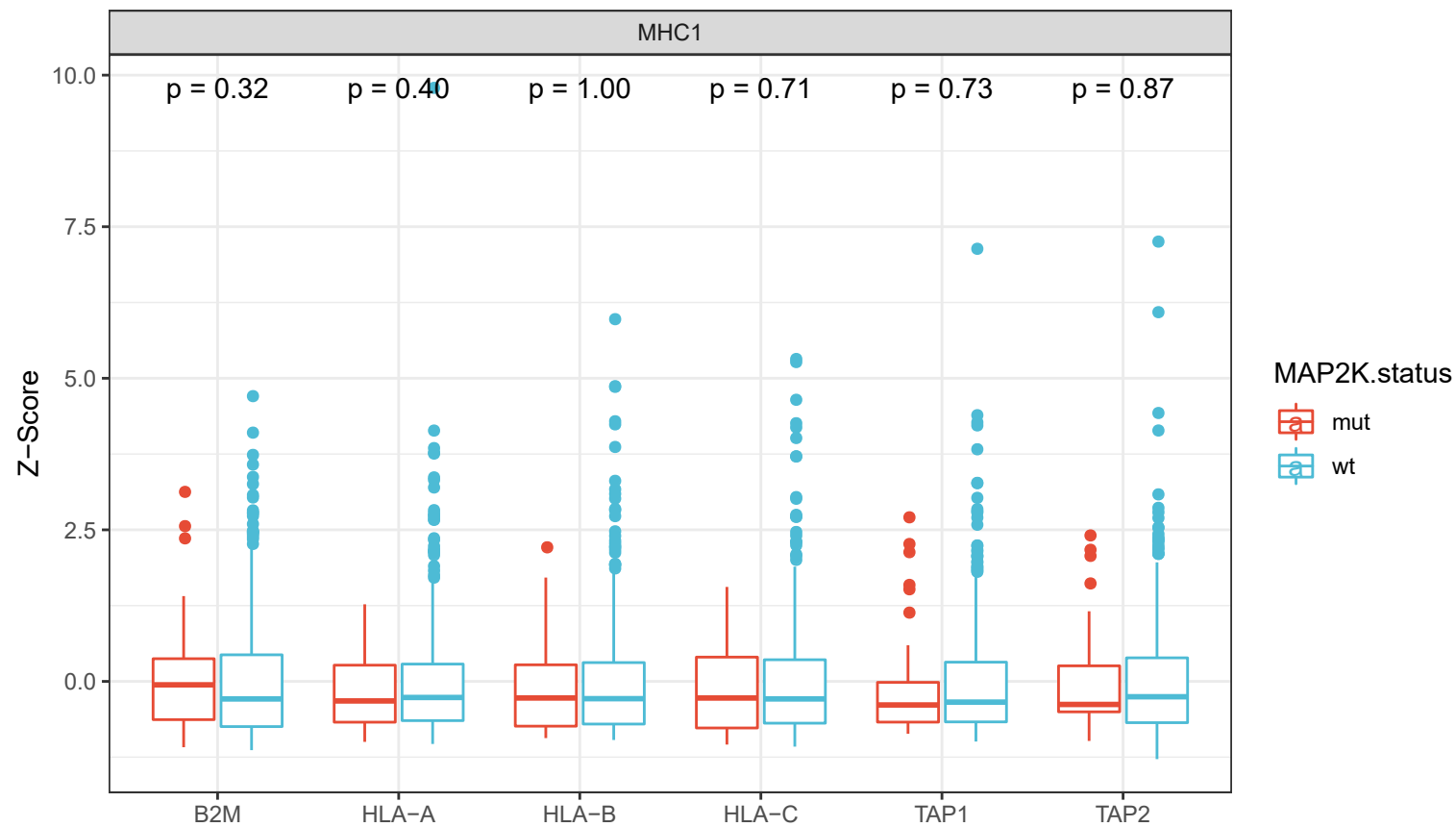

**B**

TCGA-SKCM

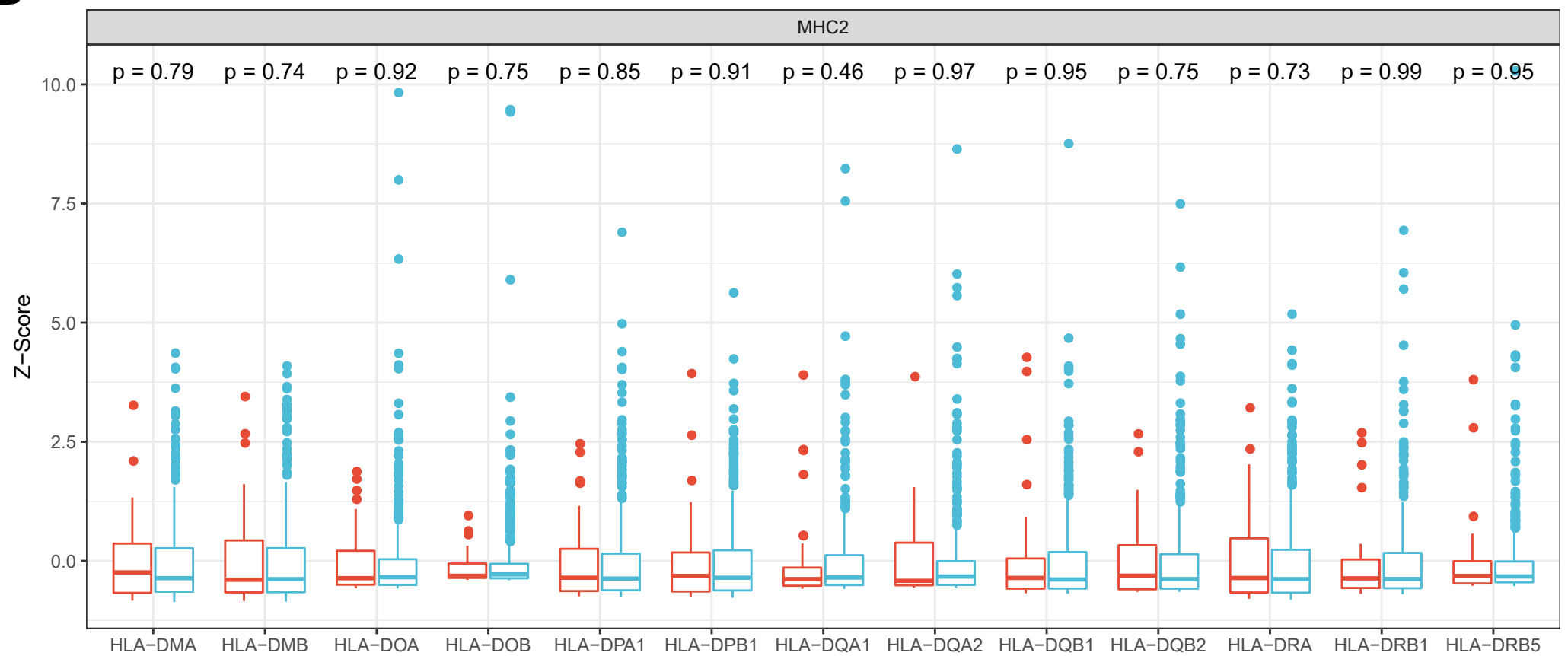

Supplement: Supplementary file 3 [file DataSheet_3.pdf]
